# Post-Covid-19 symptoms, subjective work ability and sick leave 2 years after acute infection—results from a population-based long COVID study

**DOI:** 10.1186/s12889-025-26066-w

**Published:** 2025-12-30

**Authors:** Stefanie Braig, Raphael S. Peter, Alexandra Nieters, Hans-Georg Kräusslich, Stefan O. Brockmann, Siri Göpel, Uta Merle, Jürgen M. Steinacker, Winfried V. Kern, Dietrich Rothenbacher, EPILOC Phase 3a Study Group, Christoph Bauer, Benedict Blankenhorn, Ulrike Bopp-Haas, Nadine Conzelmann, Peter Deibert, Armin Dietz, Birgit Friedmann-Bette, Veronika Götz, Sylvia Grote, Alexandra Junginger, Oliver Kappert, Anne Kühn, Lynn Matits, Barbara Müller, Andreas Nieß, Isolde Piechotowski, Sibylle Röttele, Jana Schellenberg, Claudia Schilling, Rainer Schwertz, Lisamaria Sedelmaier, Monika Spannenkrebs, Gabriele Wagner, Birgit Walter-Frank, Kersten Wolfers, Mengyu Zhu

**Affiliations:** 1 Institute of Epidemiology and Medical Biometry, Ulm University, Helmholtzstraße 22, 89081 Ulm, Germany; 2 Institute for Immunodeficiency, Medical Centre and Faculty of Medicine, Albert-Ludwigs-University, Freiburg, Germany; 3 Department of Infectious Diseases, Virology, Heidelberg University, Heidelberg, Germany; 4 Department of Health Protection, Infection Control and Epidemiology, Ministry of Social Affairs, Health and Integration, Baden-Wuerttemberg Federal State Health Office, Stuttgart, Germany; 5 Department of Internal Medicine I, University Hospital Tübingen, Tübingen, Germany; 6 Department of Internal Medicine IV, University Hospital Heidelberg, Heidelberg, Germany; 7 Division of Sports and Rehabilitation Medicine, Department of Medicine, Ulm University Hospital, Ulm, Germany; 8 Institute of Rehabilitation Medicine Research, Ulm University, Ulm, Germany; 9 Division of Infectious Diseases, Department of Medicine II, Medical Centre and Faculty of Medicine, Albert-Ludwigs-University, Freiburg, Germany

**Keywords:** SARS-CoV-2, Post-COVID syndrome, Work ability, Working task, WAI

## Abstract

**Background:**

The post-COVID syndrome (PCS) is associated with reduced work ability, increased sick leave and delayed return to work. Yet, the relationship is complex due to a heterogeneous set of PCS symptoms and the multifaceted nature of work ability.

**Methods:**

Based on a population-based longitudinal study (*n* = 5422, 18–65 years) conducted in the Southwest of Germany, we describe the evolution of work ability (mWAI1), task-related work ability (mWAI2), and sick leave 6–12 and 24 months after a SARS-CoV-2 index infection and confirmed by Polymerase Chain Reaction. Descriptive analyses on mWAI1 and mWAI2 and adjusted linear regression analyses were performed.

**Results:**

1.1% of our population was continuously on sick leave since the initial SARS-CoV-2 infection (about 24 months after the infection). Pre-infection mWAI1 was not regained due to persisting or newly occurring symptoms of fatigue, neurocognitive impairment and anxiety/depression/sleep disorders that were related also to lower mWAI2. Effect modifiers of the associations between risk factors and mWAI1 or mWAI2 were age, working tasks, and comorbid mental conditions. Further SARS-CoV-2 infections were associated with poorer mWAI2 in physically (regression coefficient, 95% confidence intervals: -3.45 (-6.15,-0.74) but not mentally working participants (0.20 (-0.54,0.95)) and age proved to be a stronger risk factor for mWAI2 in physically working subjects.

**Conclusions:**

We confirmed known risk factors but further emphasized effect modifiers like working task or comorbid mental disorders for work ability and described variables related to sick leave after SARS-CoV-2 infection.

**Supplementary Information:**

The online version contains supplementary material available at 10.1186/s12889-025-26066-w.

## What is already known on this topic

- Risk factors for reduced work ability years after a SARS-Cov-2 infection have been identified.
- Potential effect modifiers on the association between risk factors and work ability have rarely been considered.

## What this study adds

- Poor work ability is more prevalent in subjects with physical tasks compared to mental tasks and in subjects with comorbid mental disorders.
- Poor work ability is related to increased sick leave.
- Post-COVID symptoms are the main drivers for poor work ability. Fatigue and neurocognitive impairment showed the strongest associations. For sick leave, chest symptoms and mental disorders are additional risk factors.

## How this study might affect research, practice or policy

- Our results might help to identify affected subgroups that require special attention and that might be especially targeted in rehabilitation approaches.

## Introduction

Post-acute sequelae of acute SARS-CoV-2 infection have significant implications for individual well-being and societal functioning including financial disruption [1]. While the acute phase of SARS-CoV-2 infection is well described, the longer-term consequences of recovery with respect to work ability are poorly understood yet. A considerable portion of individuals with acute infection showed at least moderate impairment of daily life and work ability 6–9 months later [2] and these continued for more than a year [3].

According to the World Health Organization, a post-COVID-19 condition or post-COVID-19 syndrome (PCS) can be considered when symptoms occur within 3 months after a confirmed or probable SARS-CoV-2 infection, are not explained by other diagnoses, and persist for at least 2 months [4]. In March 2023, an estimated 1.9 million people in the UK (2.9% of the population) suffered from self-reported PCS [5]. Viral persistence, immune dysregulation, mitochondrial dysfunction, complement dysregulation, prothrombotic inflammation, and altered intestinal microbiome are being discussed as causative mechanisms (see e.g. [6], for an overview). The pathophysiology of PCS, however, is still unclear, and it is unlikely that a single mechanism can explain the varying incidence and heterogeneous set of symptoms [7].

It is estimated that COVID-19-related absence has reduced the U.S. workforce by approximately 500,000 people (0.2 percent of adults) through June 2022 [8]. Notably, in a study from Switzerland, 1.6% of the employees completely dropped out of the workforce, and 5.8% reported occupational changes due to PCS [9]. According to a review [10], fatigue, depressive symptoms, and cognitive impairment following a SARS-CoV-2 infection are associated with reduced work ability. Sociodemographic factors such as older age, being female, having a history of sick leave prior to infection, or having received inpatient care also contributed to diminishing occupational functioning [11]. Similarly, employees unable to work reported more fatigue, stroke, anxiety or depression symptoms, musculoskeletal, or chronic lung conditions [12]. Furthermore, it was emphasized that a history of a psychiatric diagnosis is associated with reductions in work ability due to PCS [13].

Work ability reflects the dynamic interplay between an individual’s internal resources and the external demands of their occupation, encompassing physical and mental health, task structure and content, and organizational conditions [14]. The Work Ability Index (WAI) is a validated instrument with acceptable test–retest reliability [15], cross-cultural applicability, and proven sensitivity to changes over time [16]. Its reduction is driven by a complex interaction of personal capacities, work-related tasks, and individual values [17, 18]. The influence of distinct PCS phenotypes on WAI remains unclear; associated symptoms may affect subgroups differently, and their relevance can vary depending on workplace context and job characteristics [13]. Furthermore, trajectories of recovery and return-to-work patterns across patient strata are still insufficiently understood.

We assessed self-reported work ability over 24 months post-COVID and analyzed subgroup trajectories considering potential effect modifiers to inform targeted rehabilitation.

## Methods

### Study design and study population

EPILOC (Epidemiology of Long COVID) is a population-based longitudinal observational study conducted in Baden-Württemberg (Southwestern Germany). The baseline assessment was conducted in 2021 and enrolled subjects between 18 and 65 years with PCR-confirmed SARS-CoV-2 index infection between 10/2020 and 04/2021. Details of the baseline assessment have been reported [2, 19]. Of the participants from the baseline study, 8813 who had given consent to be re-contacted were re-assessed in November 2022. The participation rate of the first assessment was 23.2%, of those, 61.5% also participated in the follow-up. The study was conducted according to the Declaration of Helsinki, and ethical approval was obtained of the University of Freiburg (21/1484) and Ulm University (337/21). Every participant gave written informed consent.

### Data source and measurements

The baseline questionnaire included questions on sociodemographic information, smoking, pre-existing comorbidity, height, and weight. Baseline and follow-up questionnaires (administered 6–12 and 24 months after the initial infection) contained identical questions about (additional) SARS-CoV-2 infections and a list of 30 symptoms that might have occurred before and during the index infection and at the time of answering the follow-up questionnaires. The impairment of these symptoms regarding daily living and general health was assessed on a 4-point Likert scale (none, light, moderate, strong). The reported symptoms were considered as PCS-related only if they had not been present before the acute index SARS-CoV-2 infection and if the degree of impairment was at least moderate or strong. For follow-up, both persistent and newly occurring symptoms were considered. Furthermore, a German translation of the DSQ-PEM questionnaire was used to assess post-exertional malaise (PEM), and to screen for possible Myalgic Encephalomyelitis/Chronic Fatigue Syndrome (ME/CFS) [20].

### Modified work ability index (mWAI) and work-related factors

The primary outcome was the self-reported current work ability assessed with the short-form of the Work Productivity and Activity Impairment index questionnaire [14, 21]. This WAI questionnaire was adapted to the COVID-19 situation through the following wording: “What percentage of your original working capacity (before your infection) have you regained?” with response options ranging from 0 to 100% in 10% increments (modified WAI1: mWAI1). MWAI1 was categorized into poor (≤ 60%), moderate (70–80%), and excellent (≥ 90%), as suggested [9]. Participants were asked about their working tasks (mainly physical, mainly mental, roughly equally mental and physical) and were then asked to assess their current work ability in relation to these tasks using a 5-point Likert-scale (very good, good, moderate, poor, very poor). Specifically, they responded to:”How would you assess your work ability according to your physical tasks now?”, and separately, “How would you assess your work ability according to your mental tasks now?” A score (mWAI2) was calculated by weighting the work ability according to the nature of the tasks performed. Specifically, for employees with predominantly physical tasks, work ability in relation to the physical tasks was multiplied by 1.5, work ability in relation to mental tasks by 0.5 and both values were summarized as recommended [18]. For employees with predominantly mental work the reverse weighting was applied. An equal weighting was used for participants whose work involved both physical and mental tasks in equal measure. For description and stratification, the categories of working task were collapsed into physical and mental (the latter includes mainly mental and roughly equally mental and physical). We collected baseline information on mWAI2 with reference to the period prior to SARS-CoV-2 infection, at 6–12 months, and at 2 years (included in the 24-month follow-up). For the regression analyses presented here we focus on mWAI2 measured at the 24-month follow-up. At baseline, we asked for pre-existing comorbidities, a question which is also part of the original WAI questionnaire.^.^A potential association of the following factors with WAI is discussed (see review [17]): sex, age, socioeconomic status, smoking, and obesity. We did not consider marital status as no association with mWAI1 was observed in preliminary analyses. The questionnaire included further questions on occupational status (prior to infection, at baseline and follow-up), current sick leave, change of working hours, its reason and the direction of change.

We focused on symptom clusters we had previously identified using data from the baseline assessment [2]. These clusters combined strongly correlated symptoms. Symptom clusters with the most frequent symptoms at baseline (i.e., fatigue, neurocognitive impairment, chest symptoms, smell or taste disorder, anxiety/depression/sleep disorders, musculoskeletal pain) with moderate or strong impairment, were considered. We applied the same clusters to symptoms newly or repeatedly reported 24 months after the index infection.

### Statistical methods

The characteristics of the study population with available data at 6–12 and 24 months are presented. Prevalences of mWAI1 categories and mean sick leave at 24 months are shown for the total population (main text) and for subgroups stratified by age, working task, and comorbidities (Additional file 1). Stratification by sex was additionally performed and is presented in the Additional file. The incidence rate of a change in working hours due to health and the geometric mean of sick leave weeks per person-year were calculated according to employment type (part-time versus (vs) full-time) to assess how health-related work limitations differ between employment categories. The trajectories of work ability are depicted graphically in total (main text) and stratified by age, working task, comorbidities, and symptom clusters (please see Additional file 1). We additionally contrasted the distributions of the most common symptom clusters and their relation to sick leave at 6–12 and at 24 months. The overlap of the three most common symptom clusters is displayed. Finally, adjusted linear regression models were calculated to estimate the association of risk factors with mWAI2 (measured at 24 months) and sick leave. Models included sex, educational status, current smoking, obesity, physical working task, medical treatment during acute infection, the newly occurring symptom clusters (i.e., fatigue, neurocognitive impairment, chest symptoms, musculoskeletal pain, anxiety/depression/sleep disorders, smell/taste measured at 24 months) and pre-existing comorbidities. The relationship between PEM, suspected ME/CFS and mWAI2 was determined, adjusted for confounders. Multicollinearity was excluded given a variance inflation factor < 5. We evaluated effect modification using multiplicative interaction models in linear regression analyses without further adjustment. Statistically significant interactions were observed (at *p* < 0.10) for age groups < 40 years vs ≥ 40 years, mental vs physical task, and comorbid mental disorders. SAS (release 9.4 SAS Institute Inc.) and R version 4.3.2 were used.

## Results

In total *n* = 5422 participants had information on mWAI1 at baseline and at follow-up. The median time between SARS-CoV-2 index infection and baseline (i.e., 6–12 months after index infection) was 8.7 months and 23.9 months until follow-up. The mean age at baseline was 46.9 years, and 60.1% of participants were female (Table 1). Most participants had a mild course of the index infection, with only 3.9% requiring inpatient treatment. About 47.0% had had at least a secondary SARS-CoV-2 infection during follow-up.

**Table 1:** Demographic information assessed 6–12 months after index infection and 24 months after index infection

	**Baseline population assessed 6–12 months after initial infection (*n* = 9572)**	**Follow-up population assessed 24 months after initial infection (*n* = 5422)**
**Variables**	**n (%)**	**n (%)**
**Sociodemographic characteristics**		
**Sex female**	5548 (58.0)	3258 (60.1)
**Age at baseline [years]**		
Mean (SD)	45.6 (12.7)	46.9 (12.5)
< 30	1528 (16.0)	747 (13.8)
30- < 40	1827 (19.1)	906 (16.7)
40- < 50	1817 (19.0)	1035 (19.1)
50- < 60	3104 (32.4)	1888 (34.8)
≥ 60	1296 (13.5)	846 (15.6)
**Marital status at baseline**		
Single	2390 (25.4)	1224 (23.0)
Married/living together	6572 (70.0)	3859 (72.4)
Living apart	320 (3.4)	180 (3.4)
Widowed	111 (1.2)	67 (1.3)
**Nationality** German	9019 (94.3)	5227 (96.5)
** ≥ 12 years of school education**	4629 (48.5)	2724 (50.3)
**Lifestyle**		
**Smoking status at baseline**		
Current	992 (10.4)	443 (8.2)
Former	2527 (26.5)	1457 (26.9)
Never	6029 (63.1)	3508 (64.9)
**Obesity** (BMI > 30 kg/m^2^) at baseline	1877 (19.8)	1021 (18.9)
**COVID-19-related factors**		
**Time since index infection [months]**		
Median (IQR)	8.7 (7.5–9.7)	23.9 (22.9–25.5)
**Medical treatment during COVID-19 index infection**		
None	7236 (76.3)	4047 (75.1)
Outpatient treatment	1906 (20.1)	1129 (21.0)
Inpatient treatment	273 (2.9)	160 (3.0)
Admission to ICU	73 (0.8)	50 (0.9)
**Further SARS-CoV-2-infection**	-	2515 (47.0)
**Post-Exertionelle-Malaise (PEM) or suspected ME/CFS a at 24 months**		
None	-	4241 (78.5)
PEM (but no suspected ME/CFS)	-	917 (17.0)
Suspected ME/CFS	-	248 (4.6)
**Work-related factors**		
**Occupational tasks at baseline**		
Mental/mental and physical	8492 (90.4)	4898 (91.6)
Mainly physical	899 (9.6)	449 (8.4)
**Sick leave since index SARS-CoV-2 infection**		
None	5220 (96.0)	5134 (95.2)
Continuously	84 (1.5)	60 (1.1)
Again	135 (2.5)	201 (3.7)
**Weeks of sick leave at 24 months** (Geometric Mean, 95% CI)	-	1.11 (1.10, 1.13)
**Occupation prior to SARS-CoV-2 or at follow-up**		
Full-time	3381 (62.7)	2920 (53.9)
Part-time	2012 (37.3)	1946 (35.9)
Not employed	-	216 (4.0)
Retired due to age*	-	235 (4.3)
Training and education	-	100 (1.9)
**Working hours changed due to health**		
No	-	4093 (93.0)
Yes	-	307 (7.0)
**mWAI1 [%] at the respective time point** (Mean, SD)	89.1 (16.6)	88.0 (17.5)
Median (IQR)	100 (80–100)	90 (80–100)
**mWAI2 pre-infection (%, Mean, SD)**	90.4 (10.9)	-
Median (IQR)	95 (80–100)	-
**mWAI2 (%, Mean, SD)**	79.1 (18.2)	79.9 (17.0)
Median (IQR)	80 (70–100)	80 (70–100)

*BMI* Body Mass index, *CI* Confidence interval, *IQR* Interquartile range, *ICU* Intensive care unit, *ME/CFS* Myalgic Encephalomyelitis/Chronic Fatigue Syndrome, *PCS* Post-COVID-Syndrome, *SD* Standard deviation, *mWAI1* Regained work ability after initial infection, *mWAI2* Task-related work ability

^*^if a participant was not working and was aged ≥ 60 years, the participant was treated as retired due to age

Physically working participants (8.4%) were underrepresented. At 24 months, 1.1% remained on sick leave and 3.7% reprted recurrent sick leave. The mean current work ability regained after the initial infection (mWAI1) was (self-)reported to be 89.1% (Standard deviation (SD) = 16.6) at baseline and 88.0% (SD = 17.5) at follow-up. The values for task-related work ability (prior to the infection, at baseline, and at follow-up) were 90.4% (SD = 10.9), 79.1% (SD = 18.2), and 79.9% (SD = 17.0).

**Fig. 1:**
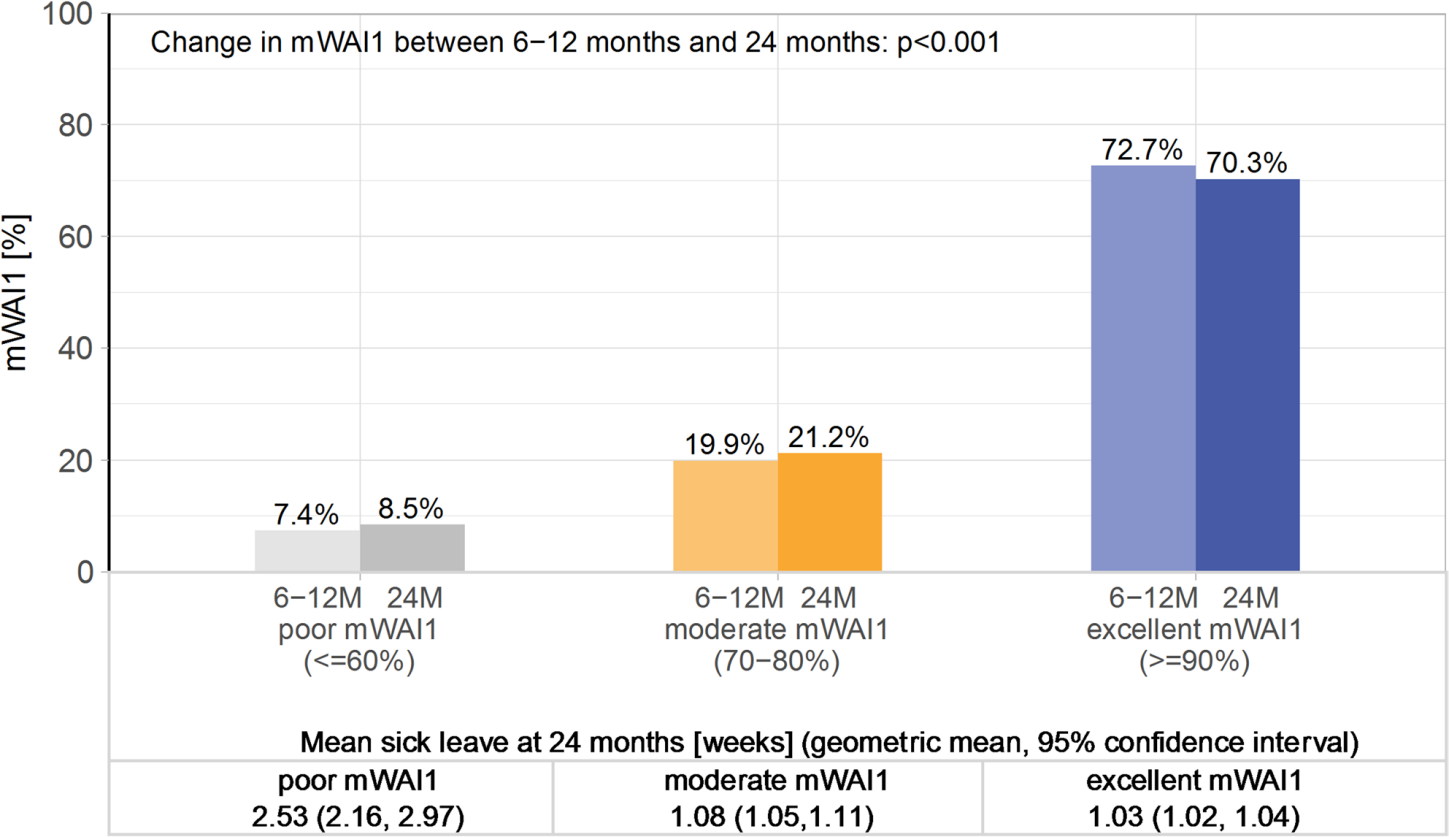
Prevalence of regained work ability at 6–12 and 24 months after initial infection (mWAI1, categorized) and mean weeks of sick leave at follow-up after 24 months in total population (*n* = 5422). The lighter shades symbolize 6–12 months the darker shades 24 months. 6-12M: 6–12 months, 24M: 24 months, mWAI1: regained modified work ability

Categorized mWAI1, as displayed in Fig. 1, emphasizes the loss of self-reported work ability after index infection, with 7.4% of participants having a poor regained mWAI1 at baseline (≤ 60%) and 8.5% at follow-up. In participants with poor mWAI1 about 24 months after infection, mean duration of sick leave (geometric mean) was 2.53 weeks (95% confidence interval (CI) 2.16, 2.97); 1.08 weeks (95% CI 1.05, 1.11) in those with moderate mWAI1, and 1.03 weeks (95% CI 1.02, 1.04) in those who rated mWAI1 as excellent. After stratification (Additional file 1, Supplementary Fig. 1), a statistically significant lower mWAI1 at follow-up was observed in the older age group and in participants without comorbid mental disorders. Distinct mWAI1 patterns emerged between mental and physical task groups.

Incidence rate of health-related working hours change was 6.4% (95% CI: 5.5%, 7.4%) in full-time employees vs 8.4% (95% CI: 6.9%, 9.8%) in part-time employees per person-year. The geometric mean of sick leave was 1.12 weeks (95% CI: 1.09, 1.14) and 1.11 weeks (95% CI: 1.08, 1.13), respectively (data not shown).

**Fig. 2:**
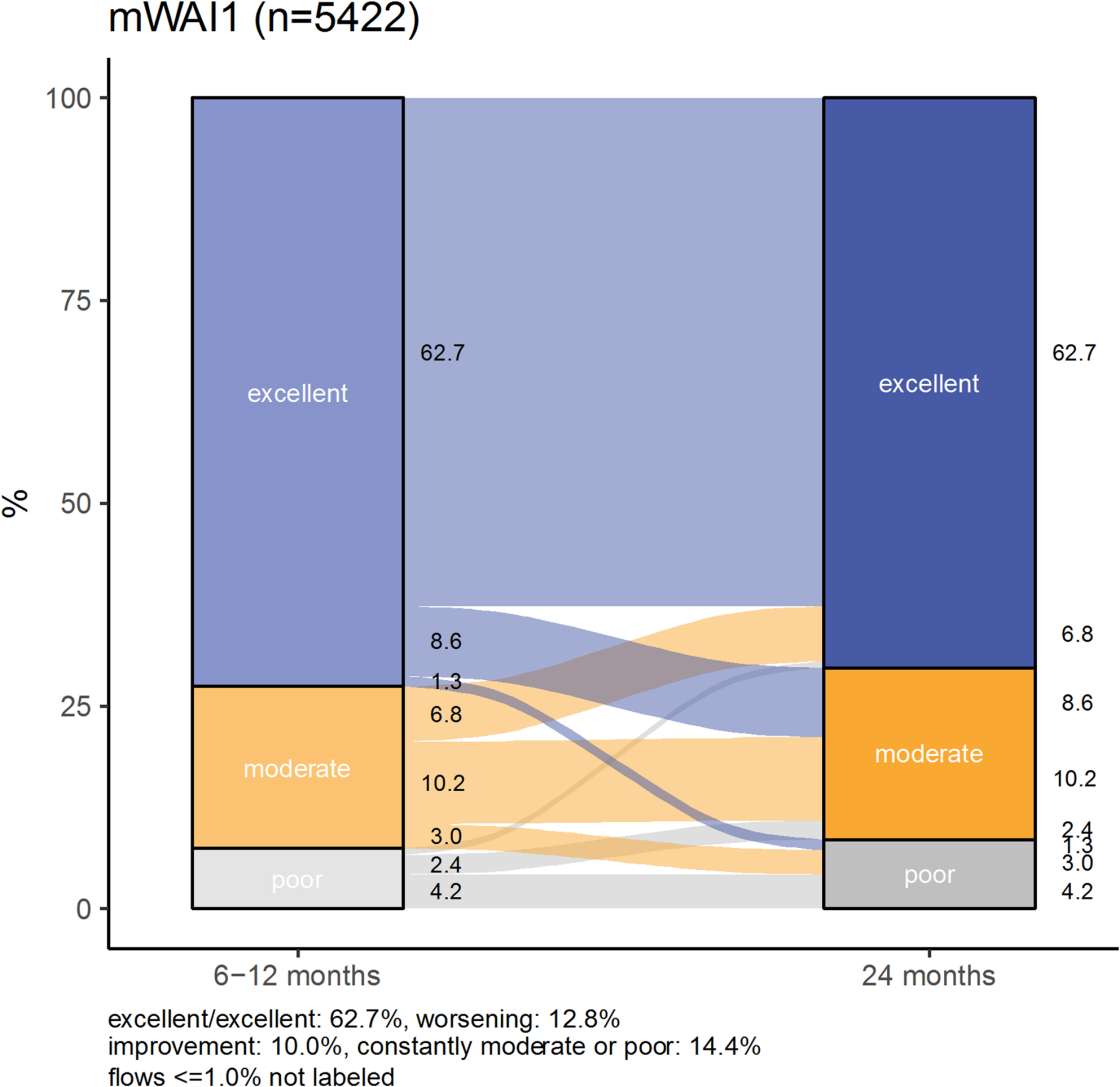
Trajectories of work ability (mWAI1, categorized) *n* = 5422, 6–12 months and 24 months after initial SARS-CoV-2 infection. mWAI1: regained modified work ability

Only 62.7% of the study population reported excellent regained work ability at 6–12 months and 24 months after the initial infection (Fig. 2). A reduction in unimpaired work ability between the two time points was observed in approximately 10.0%, while 14.4% reported “moderate or poor” mWAI1 at both time points. For the trajectories according to age, working tasks, and comorbid mental disorders please see Additional file 1, Supplementary Fig. 2. Further figures in Additional file 1 show the sex-stratified trajectories of mWAI1 according to these categories (please see Additional file 1, Supplementary Fig. 2).

**Fig. 3:**
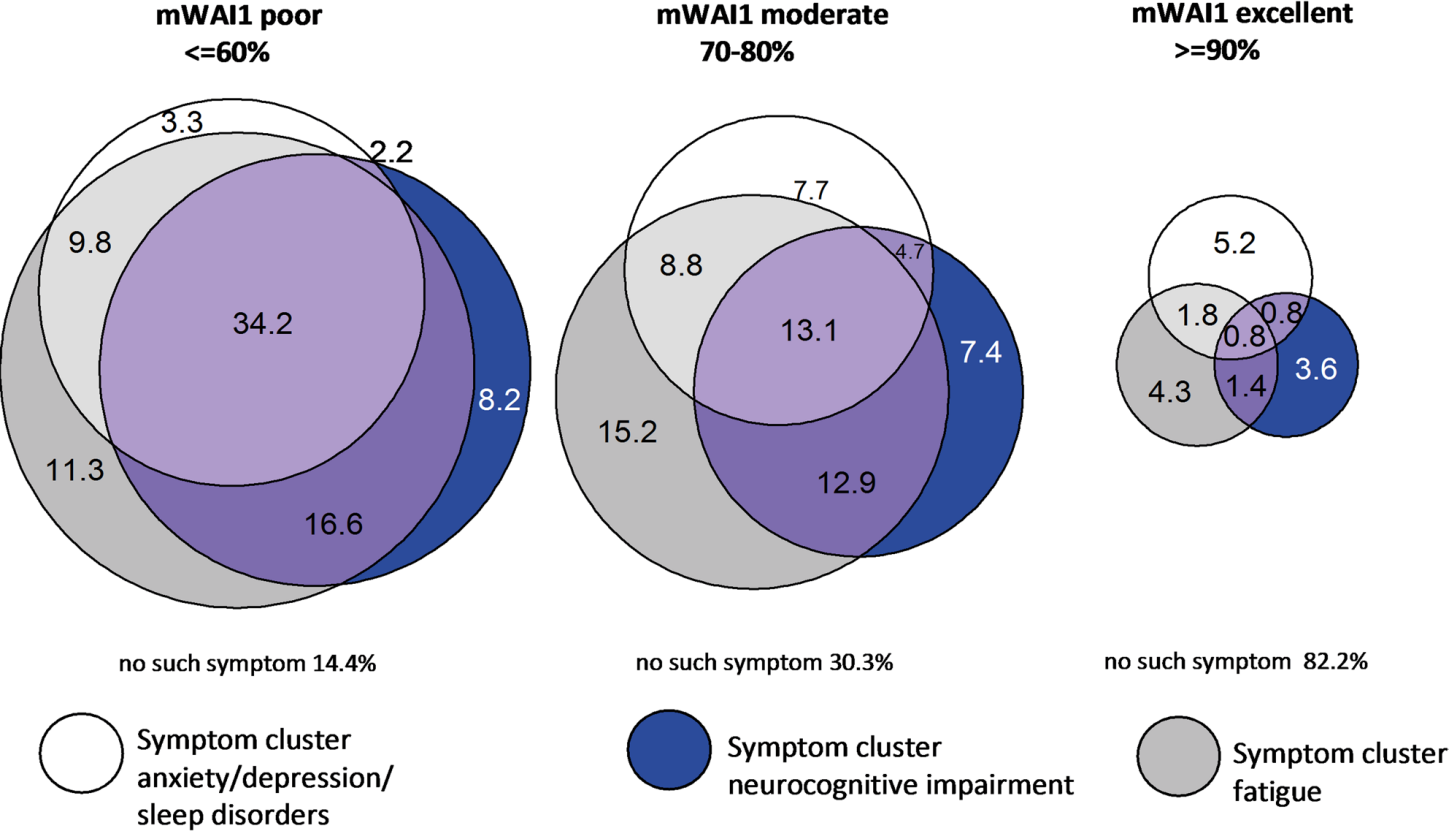
Co-occurrence of the three most prevalent symptom clusters 24 months after infection according to work ability categories (mWAI1 24 months after infection). mWAI1: regained modified work ability, numbers in the graph are %

Additional file 1, Supplementary Table 1 displays six of the most prevalent symptom clusters at baseline, their prevalence at baseline and follow-up and the duration of sick leave (assessed at follow-up). The net prevalence of the symptom clusters only changed marginally (smell/taste: −4.6%, fatigue: −2.0%, chest symptoms: −0.4%, neurocognitive impairment: + 1.4%, musculoskeletal pain: + 5.2%, anxiety/depression/sleep disorders: + 5.3%, ordered by magnitude of the change). With a geometric mean of 1.24 (95% CI 1.14, 1.34) and 1.50 weeks (95% CI 1.34, 1.59) of sick leave, the shortest time spent unable to work was in participants who reported symptoms belonging to the cluster smell/taste, while the longest was in those with chest symptoms. Trajectories of mWAI1 according to symptom clusters are displayed in Additional file 1, Supplementary Fig. 3. Still, it must be considered that symptom clusters often co-occurred, particularly in participants with poor mWAI1 or those on constant sick leave since the initial infection (Fig. 3 and Additional file 1, Supplementary Fig. 4 for sick leave).

The mutually adjusted association between potential risk factors and mWAI2 is shown in Additional file 1, Supplementary Table 2. Older age, low school education, smoking, obesity, physical working tasks, and medical treatment during index infection were all associated with a lower mWAI2. This was also true for all considered symptom clusters present at 24 months, among them fatigue and neurocognitive impairment, which showed the strongest negative association.

Stratified by age (Additional file 1, Supplementary Table 3A), female sex was associated with lower mWAI2 in participants < 40 years, but not in those ≥ 40 years (regression coefficient (b) = −1.46 95% CI −2.74, −0.18 vs b = −0.04 95% CI −0.94, 0.86). This was also true for current smoking (b = −3.28% CI −5.44, −1.13 (younger participants) vs b = −0.92 95% CI −2.57, 0.73 (older participants)). Physical working task was shown to be a risk factor for mWAI2 in older (b = −3.08 95% CI −4.68, −1.48) but not in younger participants (b = −0.30 95% CI −2.65, 2.04). Additional important factors for low mWAI2 were symptoms belonging to the symptom cluster fatigue for both age groups (b = −9.43 95% CI −11.41, −7.45 and b = −8.31 95% CI −9.61, −7.01) and neurocognitive impairment (b = −7.96 95% CI −10.03, −5.89 and b = −10.14 95% CI −11.46, −8.81). Anxiety/depression/sleep disorders were statistically significantly associated with lower mWAI2 (b = −6.18 95% CI −8.18, −4.18 and b = −6.09 95% CI −7.35, −4.84). Comorbid mental disorders, in particular, were linked to reduced mWAI2 (b = −6.70 95% CI −8.50, −4.90) in younger participants vs (b = −7.57 95% CI −8.80, −6.34) in older participants.

Additionally, older age was a risk factor for mWAI2 in mentally working (b = −2.20 95% CI −3.05, −1.35), but more pronounced in physically working participants (b = −5.25 95% CI −8.29, −2.20, Additional file 1, Supplementary Table 3D). Of note, further SARS-CoV-2 infections were statistically significantly related to lower mWAI2 in physically working participants only.

Furthermore, female sex was positively associated with mWAI2 in participants with comorbid mental disorders (b = 3.37 95% CI 1.10, 5.64, Additional file 1, Supplementary Table 3G) but negatively in those without this comorbidity (b = −1.18 95% CI −1.94, −0.41). Additional file 1, Supplementary Table 4 displays the associations between potential risk factors and weeks of sick leave. Among the findings, female sex (b = −0.78 95% CI −1.36, −0.20) and further SARS-CoV-2 infections (b = −0.61 95% CI −1.17, −0.04) were statistically significant associated with fewer weeks of sick leave, while comorbid cardiovascular (b = 0.98 95% CI 0.25, 1.71) and mental disorders (b = 2.77 95% CI 1.97, 3.57) were linked to an extended period. In contrast, symptom clusters reported 24 months post-infection—particularly fatigue (b = 2.74 95% CI 1.89, 3.60), musculoskeletal pain (b = 2.43 95% CI 1.52, 3.35) and neurocognitive impairment (b = 1.45 95% CI 0.57, 2.33)—showed the strongest positive associations with sick leave duration. Other factors such as age, education, smoking, obesity, and type of working tasks did not show statistically significant effects. Finally, we found in mutually adjusted models strong associations between PEM (b = −20.69 95% CI −21.66,−19.72), suspected ME/CFS (b = −21.14 95% CI −23.19,−19.08) and reduced mWAI2, which is described in the Additional file 1, Supplementary Table 5.

## Discussion

In our population-based cohort of 5422 predominantly mentally working participants, assessed at about 6–12 months after a SARS-CoV-2 infection, a significant decrease in work ability was observed compared to pre-infection of which a large proportion remained even after 24 months. Post-COVID related symptoms such as fatigue, neurocognitive impairment and, to a lesser extent, anxiety/depression/sleep disorders were important risk factors for a lower overall and task-related work ability. Effect modifiers such as age, working tasks, and comorbid mental disorders were identified, knowledge which may enable more tailored interventions for high-risk groups.

In our population-based cohort study, 1.1% of participants had been continuously on sick leave since the initial infection, while 3.7% experienced recurrent sick leave at 24 months. According to data from a German health insurance company, approximately 0.4% of employees were wholly or partially withdrawn from the labour market due to PCS [22]. Similarly, over a 30-month period, PCS burden decreased, but 1% still experienced significant restrictions in their daily lives [23]. In our study, a proportion of 4.0% were not employed at follow-up, highlighting the potential economic burden of PCS. Variation in labour market withdrawal across studies likely reflects differences in populations and work environments.

According to the Federal Statistical Office of Germany [24], 25% of employed individuals aged 15–74 spent at least half their working time doing physically demanding tasks, with higher rates among men (28%) than women (21%) – diverging from our findings as we observed a lower overall proportion of employees with physical tasks. Physical work is more common among those with lower educational attainment and among migrants: 35% of employed migrants – a demographic group that was insufficiently captured in our sample – perform physically strenuous labour compared to 22% of non-migrants. These disparities likely also reflect differences in educational attainment of migrants who are often disproportionately affected by lower levels of formal education or by the non-recognition of qualifications obtained in their countries of origin. Consequently, if our study population more accurately mirrored the demographic composition of the general population, the observed prevalence of low mWAI might be higher.

Our results on sex and mWAI2 were inconclusive as we observed a positive association between female sex and mWAI2 in participants with mental comorbidity, while also noting a negative association with duration of sick leave. This contrasts with previous research, which has shown that women are twice as likely to develop PCS as men [25] with potential effects on work ability. It also differs from previous studies on return-to-work outcomes (e.g., [26] or review: Ottiger and colleagues [10]). We suggest that our simultaneous adjustment for symptoms and comorbid medical conditions may have contributed to the positive association and consider whether over-adjustment might be a factor. However, our findings may also highlight the significant role of mental illness in the link between PCS and work disability. Interestingly, in a Swedish cohort, the cumulative incidence of 12-month sick leave following a SARS-CoV-2 infection was 3.5% in men and 2.7% in women, although women tended to experience longer sick leave durations [27].

Smoking is associated with an increased risk of developing PCS (see review: Trofor and colleagues [28]) and with later return to work in PCS patients [29]. In our data, however, smoking affected work ability primarily in younger individuals with mental tasks/comorbidity; other determinants, such as age and further comorbidities, aligned with existing evidence [30]. Notably, after mutual adjustment (and stratification), the impact of hospitalization during the acute infection was attenuated in our study.

Moreover, further SARS-CoV-2 infections showed no association with mWAI2 in most of our analyses, except among physically working participants. This aligns with data suggesting that hybrid immunity may considerably decrease the risk of PCS [31]. However, physically working participants may differ from mentally working subjects, e.g., in their ability to regulate their workload.

The central role of fatigue in PCS symptoms is well described [27, 32]. Moreover, it was highlighted that, despite rehabilitation, PCS symptoms such as fatigue, neurocognitive impairments, and muscle/joint pain persisted, whereas dyspnea and chest pain showed significant improvement [33, 34]. Our findings confirmed the detrimental impact of fatigue and neurocognitive impairment, with the latter being the most harmful. However, among physically working participants, we also observed a pronounced reduction in mWAI2 associated with chest symptoms (particularly in male participants), highlighting the need for targeted interventions. Likewise, individuals with comorbid mental disorders warrant special focus, as only 35.2% reported excellent mWAI1 at both time points (17.7% constantly moderate mWAI1, 13.2% constantly poor mWAI1) compared to 69.9% (8.3%, 1.9%) of those without such conditions (Additional file 1, Supplementary Fig. 2) with no strong influence of sex observed.

The strengths of our work are the population-based longitudinal design in defined geographical regions, the large sample size, and the long follow-up period of 24 months (median). Limitations include the lack of medical validation of self-reported symptoms and incomplete information on infections other than SARS-CoV-2. Our data mainly included wild-type infected individuals with the highest disease burden [23]. Importantly, also among uninfected individuals who never experienced COVID-19, 21.2% reported long COVID-compatible symptoms [35]. Therefore, misclassification cannot be ruled out. Also, selective response bias has to be considered.

Taken together, our study confirms known risk factors for reduced post-COVID work ability and highlights additional subgroup-specific risks, suggesting targeted rehabilitation strategies.

## Supplementary Information

Additional file 1. Supplementary Figure 1: Prevalence of regained work ability at 6-12 and 24 months after initial infection (mWAI1, categorized) and mean weeks of sick leave according to age, working task, and comorbid mental disorders. The lighter shades symbolize 6-12 months the darker shades 24 months. 6-12M: 6-12 months, 24M: 24 months, mWAI1: regained modified work ability. Supplementary Figure 2: Trajectories of work ability (mWAI1, categorized) in *n*=5422 participants, 6-12 months and 24 months after infection according to age, working task, and comorbid mental disorders. 6-12M: 6-12 months, 24M: 24 months, mWAI1: regained modified work ability. Supplementary Figure 3: Trajectories of work ability (mWAI1, categorized) 6-12 months and 24 months after infection according to symptom clusters newly occurred 6-12 months after initial infection. 6-12M: 6-12 months, 24M: 24 months, mWAI1: regained modified work ability. Supplementary Figure 4: Co-occurrence of the three most prevalent symptom clusters 24 months after infection according to sick leave (24 months after infection). Numbers in the graphs are %. Supplementary Table 1: Prevalence of the six most common symptom clusters at 6-12 months and at 24 months and mean time of sick leave (weeks). Supplementary Table 2: Association between potential risk factors including symptom clusters measured 24 months after initial infection and task related work ability (mWAI2 measured at 24 months). Results of a mutually adjusted linear regression analysis (*n*=5422). Supplementary Table 3A: Association between potential risk factors including symptom clusters measured 24 months after initial infection and task related work ability (mWAI2). Results of a mutually adjusted linear regression analysis stratified by age. Supplementary Table 3B: Association between potential risk factors including symptom clusters measured 24 months after initial infection and task related work ability (mWAI2). Results of a mutually adjusted linear regression analysis stratified by age among female participants. Supplementary Table 3C: Association between potential risk factors including symptom clusters measured 24 months after initial infection and task related work ability (mWAI2). Results of a mutually adjusted linear regression analysis stratified by age among male participants. Supplementary Table 3D: Association between potential risk factors including symptom clusters measured 24 months after initial infection and task related work ability (mWAI2). Results of a mutually adjusted linear regression analysis stratified by working task. Supplementary Table 3E: Association between potential risk factors including symptom clusters measured 24 months after initial infection and task related work ability (mWAI2). Results of a mutually adjusted linear regression analysis stratified by working task among female participants. Supplementary Table 3F: Association between potential risk factors including symptom clusters measured 24 months after initial infection and task related work ability (mWAI2). Results of a mutually adjusted linear regression analysis stratified by working task among male participants. Supplementary Table 3G: Association between potential risk factors including symptom clusters reported 24 months after initial infection and task related work ability (mWAI2). Results of a mutually adjusted linear regression analysis stratified by comorbid mental disorders. Supplementary Table 3H: Association between potential risk factors including symptom clusters reported 24 months after initial infection and task related work ability (mWAI2). Results of a mutually adjusted linear regression analysis stratified by comorbid mental disorders among female participants. Supplementary Table 3I: Association between potential risk factors including symptom clusters reported 24 months after initial infection and task related work ability (mWAI2). Results of a mutually adjusted linear regression analysis stratified by comorbid mental disorders among male participants. Additional file 1, Supplementary Table 4: Association between potential risk factors including symptom clusters reported 24 months after initial infection and weeks of sick leave. Results of a mutually adjusted linear regression analysis (*n *= 5422). Supplementary Table 5: Association between potential risk factors including post-exertional malaise or ME/CFS and task related work ability (mWAI2). Results of a mutually adjusted linear regression analysis (*n *= 5422). Supplementary Table 2: mWAI2: modified work ability index, task related work ability, Symptom clusters were not present before the SARS-CoV-2 infection, bold letters indicate statistically significant at *p *< 0.05. Supplementary Table 3A: mWAI2: modified work ability index, task related work ability, Symptom clusters were not present before the SARS-CoV-2 infection, bold letters indicate statistically significant at *p *< 0.05. Supplementary Table 3B: mWAI2: modified work ability index, task related work ability, Symptom clusters were not present before the SARS-CoV-2 infection, bold letters indicate statistically significant at *p *< 0.05.Supplementary Table 3C:mWAI2: modified work ability index, task related work ability, Symptom clusters were not present before the SARS-CoV-2 infection, bold letters indicate statistically significant at *p *< 0.05. Supplementary Table 3D:mWAI2: modified work ability index, task related work ability, Symptom clusters were not present before the SARS-CoV-2 infection, bold letters indicate statistically significant at *p *< 0.05. Supplementary Table 3E: mWAI2: modified work ability index, task related work ability, Symptom clusters were not present before the SARS-CoV-2 infection, bold letters indicate statistically significant at *p *< 0.05. Supplementary Table 3F:mWAI2: modified work ability index, task related work ability, Symptom clusters were not present before the SARS-CoV-2 infection, bold letters indicate statistically significant at *p *< 0.05. Supplementary Table 3G: mWAI2: modified work ability index, task related work ability, Symptom clusters were not present before the SARS-CoV-2 infection, bold letters indicate statistically significant at *p *< 0.05. Supplementary Table 3H: mWAI2: modified work ability index, task related work ability, Symptom clusters were not present before the SARS-CoV-2 infection, bold letters indicate statistically significant at *p *< 0.05. Supplementary Table 3I: mWAI2: modified work ability index, task related work ability, Symptom clusters were not present before the SARS-CoV-2 infection, bold letters indicate statistically significant at *p *< 0.05. Supplementary Table 4: Symptom clusters were not present before the SARS-CoV-2 infection, bold letters indicate statistically significant at *p *< 0.05. Supplementary Table 5: mWAI2: modified work ability index, task related work ability, bold letters indicate statistically significant at* p *< 0.05, ME/CFS: Myalgic Encephalomyelitis/Chronic Fatigue Syndrome, #in the model with post-exertional malaise we did not adjust for ME/CFS and vice versa.

## Data Availability

The EPILOC consortium has established a data access and use committee; requests may be sent to dauc.epiloc@uni-ulm.de.

## Abbreviations

b: Regression coefficient
CI: Confidence interval
ME/CFS: Myalgic Encephalomyelitis/Chronic Fatigue Syndrome
mWAI1: Work ability index modified
mWAI2: Task-related work ability
PCR: Polymerase chain reaction
PCS: Post-COVID-19 syndrome
PEM: Post-exertional malaise
SARS-CoV-2: Severe acute respiratory syndrome coronavirus 2
SD: Standard deviation
vs: Versus
WAI: Work ability index
