# Supplementary material for: Post-Covid-19 symptoms, subjective work ability and sick leave 2 years after acute infection—results from a population-based long COVID study
Source: BMC Public Health. 2025 Dec 30;26:354. doi: 10.1186/s12889-025-26066-w (PMC12849167; doi:10.1186/s12889-025-26066-w)
Supplement: Supplementary file 1 — Additional file 1. Supplementary Figure 1: Prevalence of regained work ability at 6-12 and 24 months after initial infection (mWAI1, categorized) and mean weeks of sick leave according to age, working task, and comorbid mental disorders. The lighter shades symbolize 6-12 months the darker shades 24 months. 6-12M: 6-12 months, 24M: 24 months, mWAI1: regained modified work ability. Supplementary Figure 2: Trajectories of work ability (mWAI1, categorized) in n=5422 participants, 6-12 months and 24 months after infection according to age, working task, and comorbid mental disorders. 6-12M: 6-12 months, 24M: 24 months, mWAI1: regained modified work ability. Supplementary Figure 3: Trajectories of work ability (mWAI1, categorized) 6-12 months and 24 months after infection according to symptom clusters newly occurred 6-12 months after initial infection. 6-12M: 6-12 months, 24M: 24 months, mWAI1: regained modified work ability. Supplementary Figure 4: Co-occurrence of the three most prevalent symptom clusters 24 months after infection according to sick leave (24 months after infection). Numbers in the graphs are %. Supplementary Table 1: Prevalence of the six most common symptom clusters at 6-12 months and at 24 months and mean time of sick leave (weeks). Supplementary Table 2: Association between potential risk factors including symptom clusters measured 24 months after initial infection and task related work ability (mWAI2 measured at 24 months). Results of a mutually adjusted linear regression analysis (n=5422). Supplementary Table 3A: Association between potential risk factors including symptom clusters measured 24 months after initial infection and task related work ability (mWAI2). Results of a mutually adjusted linear regression analysis stratified by age. Supplementary Table 3B: Association between potential risk factors including symptom clusters measured 24 months after initial infection and task related work ability (mWAI2). Results of a mutually adjusted linear [file 12889_2025_26066_MOESM1_ESM.docx]

**Additional file 1:**

**Supplementary Figure 1: Prevalence of regained work ability at 6-12 and 24 months after initial infection (mWAI1, categorized) and mean weeks of sick leave according to age, working task, and comorbid mental disorders. The lighter shades symbolize 6-12 months the darker shades 24 months.** 6-12M: 6-12 months, 24M: 24 months, mWAI1: regained modified work ability.

**Total population**

**Female participants**

**Supplementary Figure 1:** …continued

**Male participants**

**Supplementary Figure 1:** …continued

**Total population**

**Female participants**

**Male participants**

**Supplementary Figure 1:** …continued

**Total population**

**Female participants**

**Male participants**

**Supplementary Figure 2:** **Trajectories of work ability (mWAI1, categorized) in n=5422 participants, 6-12 months and 24 months after infection according to age, working task, and comorbid mental disorders.** 6-12M: 6-12 months, 24M: 24 months, mWAI1: regained modified work ability.

**Total population**

**Female participants**

**Supplementary Figure 2:** …continued

**Male participants**

**Supplementary Figure 2:** …continued

**Total population**

**Female participants**

**Supplementary Figure 2:** …continued

**Male participants**

**Supplementary Figure 2:** …continued

**Total population**

**Female participants**

**Supplementary Figure 2:** …continued

**Male participants**

**Supplementary Table 1:** **Prevalence of the six most common symptom clusters at 6-12 months and at 24 months and mean time of sick leave (weeks).**

|  | **6-12 months after initial infection** | **24 months after  initial infection** | | |
| --- | --- | --- | --- | --- |
| Symptom clusters | Prevalence n (%) | Prevalence  n (%) | Net difference | Geometric mean of sick leave in weeks (95% CI)# |
| Fatigue | 1292 (24.6) | 1212 (22.6) | -2.0% | 1.45 (1.35, 1.54) |
| Neurocognitive impairment | 863 (16.4) | 957 (17.8) | +1.4% | 1.44 (1.34, 1.55) |
| Chest symptoms | 780 (14.7) | 765 (14.3) | -0.4% | 1.50 (1.34, 1.59) |
| Musculoskeletal pain | 503 (9.5) | 787 (14.7) | +5.2% | 1.46 (1.35, 1.59) |
| Anxiety/depression/sleep disorders | 642 (12.1) | 936 (17.4) | +5.3% | 1.38 (1.29, 1.48) |
| Smell/taste | 646 (12.3) | 409 (7.7) | -4.6% | 1.24 (1.14, 1.34) |
| None of the symptom clusters above | 3197 (59.8) | 3075 (56.9) | -2.9% | 1.03 (1.02, 1.04) |

CI: confidence intervals, #according to symptom clusters at 24 months

**Supplementary Figure 3: Trajectories of work ability (mWAI1, categorized) 6-12 months and 24 months after infection according to symptom clusters newly occurred 6-12 months after initial infection.** 6-12M: 6-12 months, 24M: 24 months, mWAI1: regained modified work ability.

**Supplementary Figure 3**: …continued

**Supplementary Figure 3**: …continued

**Supplementary Figure 4: Co-occurrence of the three most prevalent symptom clusters 24 months after infection according to sick leave (24 months after infection).** Numbers in the graphs are %.

**Supplementary Table 2****: Association between potential risk factors including symptom clusters measured 24 months after initial infection and task related work ability (mWAI2 measured at 24 months). Results of a mutually adjusted linear regression analysis (n=5422).**

| **Predictor** | **Estimate** | **p value** | **Lower 95% Confidence interval** | **Upper 95% Confidence interval** |
| --- | --- | --- | --- | --- |
| Sex female | -0.53 | 0.16 | -1.27 | 0.20 |
| Age >40 years | **-2.41** | **<0.001** | **-3.23** | **-1.59** |
| >= 12 years of school education | **1.86** | **<0.001** | **1.11** | **2.62** |
| Smoking | **-1.70** | **0.011** | **-3.01** | **-0.39** |
| Obesity | **-1.61** | **0.001** | **-2.57** | **-0.65** |
| Physical working task | **-2.15** | **0.001** | **-3.47** | **-0.84** |
| Medical treatment during index infection | **-1.81** | **<0.001** | **-2.69** | **-0.93** |
| Further SARS-CoV-2-infection | -0.08 | 0.82 | -0.80 | 0.64 |
| *Symptom clusters 24 months* |  |  |  |  |
| Fatigue | **-8.65** | **<0.001** | **-9.73** | **-7.57** |
| Neurocognitive impairment | **-9.51** | **<0.001** | **-10.62** | **-8.40** |
| Chest symptoms | **-3.63** | **<0.001** | **-4.84** | **-2.42** |
| Musculoskeletal pain | **-3.35** | **<0.001** | **-4.50** | **-2.19** |
| Anxiety/depression/sleep disorder | **-6.13** | **<0.001** | **-7.19** | **-5.07** |
| Smell/taste | **-2.31** | **0.001** | **-3.70** | **-0.92** |
| *Pre-existing comorbidities* |  |  |  |  |
| Musculoskeletal disorders | **-2.11** | **<0.001** | **-2.90** | **-1.32** |
| Cardiovascular diseases | **-2.24** | **<0.001** | **-3.16** | **-1.32** |
| Respiratory diseases | **-1.35** | **0.008** | **-2.34** | **-0.35** |
| Mental disorders | **-7.38** | **<0.001** | **-8.39** | **-6.36** |

mWAI2: modified work ability index, task related work ability, Symptom clusters were not present before the SARS-CoV-2 infection, bold letters indicate statistically significant at p<0.05

**Supplementary Table 3A:** **Association between potential risk factors including symptom clusters measured 24 months after initial infection and task related work ability (mWAI2). Results of a mutually adjusted linear regression analysis stratified by age.**

|  | **Total population, age <40 years (n=1653)** | | | | **Total population, age >=40 years (n=3769)** | | | | |
| --- | --- | --- | --- | --- | --- | --- | --- | --- | --- |
| **Predictor** | **Estimate** | **p value** | **Lower 95% Confidence interval** | **Upper 95% Confidence interval** | **Estimate** | **p value** | **Lower 95% Confidence interval** | | **Upper 95% Confidence interval** |
| Sex female | **-1.46** | **0.026** | **-2.74** | **-0.18** | -0.04 | 0.93 | -0.94 | 0.86 | |
| >= 12 years of school education | 0.85 | 0.23 | -0.54 | 2.24 | **2.31** | **<0.001** | **1.40** | | **3.21** |
| Current smoker | **-3.28** | **0.003** | **-5.44** | **-1.13** | -0.92 | 0.28 | -2.57 | | 0.73 |
| Obesity | **-2.09** | **0.032** | **-4.00** | **-0.18** | **-1.45** | **0.011** | **-2.56** | | **-0.33** |
| Physical working task | -0.30 | 0.80 | -2.65 | 2.04 | **-3.08** | **<0.001** | **-4.68** | | **-1.48** |
| Medical treatment during index infection | **-2.26** | **0.009** | **-3.97** | **-0.56** | **-1.62** | **0.002** | **-2.65** | | **-0.58** |
| Further SARS-CoV2-infection | -0.18 | 0.78 | -1.41 | 1.06 | -0.03 | 0.93 | -0.92 | | 0.85 |
| *Symptom clusters 24 months* |  |  |  |  |  |  |  | |  |
| Fatigue | **-9.43** | **<0.001** | **-11.41** | **-7.45** | **-8.31** | **<0.001** | **-9.61** | | **-7.01** |
| Neurocognitive impairment | **-7.96** | **<0.001** | **-10.03** | **-5.89** | **-10.14** | **<0.001** | **-11.46** | | **-8.81** |
| Chest symptoms | **-3.91** | **0.002** | **-6.32** | **-1.50** | **-3.54** | **<0.001** | **-4.94** | | **-2.14** |
| Musculoskeletal pain | **-3.27** | **0.010** | **-5.77** | **-0.78** | **-3.35** | **<0.001** | **-4.66** | | **-2.03** |
| Anxiety/depression/sleep disorders | **-6.18** | **<0.001** | **-8.18** | **-4.18** | **-6.09** | **<0.001** | **-7.35** | | **-4.84** |
| Smell/taste | -2.47 | 0.076 | -5.20 | 0.26 | **-2.27** | **0.006** | **-3.89** | | **-0.65** |
| *Pre-existing comorbidities* |  |  |  |  |  |  |  | |  |
| Musculoskeletal disorders | **-1.76** | **0.028** | **-3.33** | **-0.19** | **-2.16** | **<0.001** | **-3.08** | | **-1.24** |
| Cardiovascular diseases | -1.72 | 0.17 | -4.18 | 0.73 | **-2.27** | **<0.001** | **-3.27** | | **-1.26** |
| Respiratory diseases | -0.88 | 0.35 | -2.71 | 0.95 | **-1.51** | **0.013** | **-2.71** | | **-0.32** |
| Mental disorders | **-6.70** | **<0.001** | **-8.50** | **-4.90** | **-7.57** | **<0.001** | **-8.80** | | **-6.34** |

mWAI2: modified work ability index, task related work ability, Symptom clusters were not present before the SARS-CoV-2 infection, bold letters indicate statistically significant at p<0.05

**Supplementary Table 3B: Association between potential risk factors including symptom clusters measured 24 months after initial infection and task related work ability (mWAI2). Results of a mutually adjusted linear regression analysis stratified by age among female participants.**

|  | **Female participants, age <40 years (n=1033)** | | | | **Female participants, age >=40 years (n=2225)** | | | |
| --- | --- | --- | --- | --- | --- | --- | --- | --- |
| **Predictor** | **Estimate** | **p value** | **Lower 95% Confidence interval** | **Upper 95% Confidence interval** | **Estimate** | **p value** | **Lower 95% Confidence interval** | **Upper 95% Confidence interval** |
| >= 12 years of school education | 1.57 | 0.08 | -0.21 | 3.34 | **1.87** | **0.002** | **0.70** | **3.03** |
| Current smoker | -2.72 | 0.057 | -5.52 | 0.08 | -1.83 | 0.081 | -3.87 | 0.22 |
| Obesity | -2.19 | 0.081 | -4.65 | 0.27 | -0.30 | 0.69 | -1.76 | 1.16 |
| Physical working task | -0.62 | 0.70 | -3.81 | 2.57 | **-2.41** | **0.019** | **-4.42** | **-0.39** |
| Medical treatment during index infection | -1.41 | 0.20 | -3.55 | 0.74 | **-1.54** | **0.022** | **-2.86** | **-0.22** |
| Further SARS-CoV2-infection | -0.24 | 0.77 | -1.85 | 1.38 | 0.30 | 0.60 | -0.83 | 1.43 |
| *Symptom clusters 24 months* |  |  |  |  |  |  |  |  |
| Fatigue | **-9.43** | **<0.001** | **-11.76** | -7.10 | **-7.46** | **<0.001** | **-9.03** | **-5.89** |
| Neurocognitive impairment | **-7.11** | **<0.001** | **-9.64** | -4.59 | **-9.68** | **<0.001** | **-11.27** | **-8.09** |
| Chest symptoms | **-4.19** | **0.003** | **-7.00** | -1.39 | **-2.62** | **0.003** | **-4.33** | **-0.90** |
| Musculoskeletal pain | **-5.18** | **0.001** | **-8.20** | -2.15 | **-3.88** | **<0.001** | **-5.47** | **-2.28** |
| Anxiety/depression/sleep disorders | **-5.85** | **<0.001** | **-8.32** | -3.38 | **-4.62** | **<0.001** | **-6.16** | **-3.08** |
| Smell/taste | -0.52 | 0.75 | -3.77 | 2.72 | **-3.16** | **0.001** | **-5.09** | **-1.22** |
| *Pre-existing comorbidities* |  |  |  |  |  |  |  |  |
| Musculoskeletal disorders | 0.10 | 0.92 | -1.91 | 2.11 | **-1.29** | **0.030** | **-2.45** | **-0.12** |
| Cardiovascular diseases | -1.26 | 0.47 | -4.67 | 2.14 | **-2.55** | **<0.001** | **-3.88** | **-1.22** |
| Respiratory diseases | -0.62 | 0.59 | -2.90 | 1.66 | **-2.40** | **0.002** | **-3.90** | **-0.89** |
| Mental disorders | -5.36 | **<0.001** | -7.53 | -3.18 | **-6.79** | **<0.001** | **-8.27** | **-5.31** |

mWAI2: modified work ability index, task related work ability, Symptom clusters were not present before the SARS-CoV-2 infection, bold letters indicate statistically significant at p<0.05

**Supplementary Table 3C: Association between potential risk factors including symptom clusters measured 24 months after initial infection and task related work ability (mWAI2). Results of a mutually adjusted linear regression analysis stratified by age among male participants.**

|  | **Male participants, age <40 years (n=620)** | | | | **Male participants, age >=40 years (n=1544)** | | | |
| --- | --- | --- | --- | --- | --- | --- | --- | --- |
| **Predictor** | **Estimate** | **p value** | **Lower 95% Confidence interval** | **Upper 95% Confidence interval** | **Estimate** | **p value** | **Lower 95% Confidence interval** | **Upper 95% Confidence interval** |
| >= 12 years of school education | **0.38** | **0.74** | **-1.89** | **2.65** | **2.55** | **0.001** | **1.12** | **3.97** |
| Current smoker | **-3.96** | **0.022** | **-7.34** | **-0.58** | 0.55 | 0.69 | -2.18 | 3.28 |
| Obesity | -2.07 | 0.18 | -5.10 | 0.96 | **-3.03** | **<0.001** | **-4.75** | **-1.31** |
| Physical working task | 0.32 | 0.85 | -3.14 | 3.78 | **-3.57** | **0.007** | **-6.16** | **-0.98** |
| Medical treatment during index infection | **-3.90** | **0.008** | **-6.77** | **-1.02** | **-1.73** | **0.039** | **-3.38** | **-0.09** |
| Further SARS-CoV2-infection | -0.36 | 0.72 | -2.28 | 1.56 | -0.37 | 0.61 | -1.77 | 1.04 |
| *Symptom clusters 24 months* |  |  |  |  |  |  |  |  |
| Fatigue | **-10.19** | **<0.001** | **-14.03** | **-6.36** | **-10.27** | **<0.001** | **-12.54** | **-8.00** |
| Neurocognitive impairment | **-8.86** | **<0.001** | **-12.52** | **-5.19** | **-10.50** | **<0.001** | **-12.81** | **-8.18** |
| Chest symptoms | -3.53 | 0.17 | -8.54 | 1.48 | **-4.53** | **<0.001** | **-6.93** | **-2.14** |
| Musculoskeletal pain | -0.21 | 0.94 | -4.74 | 4.33 | **-2.43** | **0.039** | **-4.72** | **-0.14** |
| Anxiety/depression/sleep disorders | **-7.19** | **<0.001** | **-10.63** | **-3.75** | **-8.79** | **<0.001** | **-10.91** | **-6.67** |
| Smell/taste | **-6.79** | **0.011** | **-12.03** | **-1.55** | -0.86 | 0.56 | -3.76 | 2.04 |
| *Pre-existing comorbidities* |  |  |  |  |  |  |  |  |
| Musculoskeletal disorders | **-5.20** | **<0.001** | **-7.73** | **-2.67** | **-3.32** | **<0.001** | **-4.80** | **-1.85** |
| Cardiovascular diseases | -2.64 | 0.14 | -6.15 | 0.87 | **-1.76** | **0.023** | **-3.28** | **-0.24** |
| Respiratory diseases | -1.04 | 0.52 | -4.19 | 2.12 | -0.24 | 0.80 | -2.16 | 1.68 |
| Mental disorders | **-10.54** | **<0.001** | **-13.87** | **-7.21** | **-9.24** | **<0.001** | **-11.39** | **-7.10** |

mWAI2: modified work ability index, task related work ability, Symptom clusters were not present before the SARS-CoV-2 infection, bold letters indicate statistically significant at p<0.05

**Supplementary Table 3D:** **Association between potential risk factors including symptom clusters measured 24 months after initial infection and task related work ability (mWAI2). Results of a mutually adjusted linear regression analysis stratified by working task.**

|  | **Total population with mental working task (n=4898)** | | | | **Total population with physical working task (n=449)** | | | |
| --- | --- | --- | --- | --- | --- | --- | --- | --- |
| **Predictor** | **Estimate** | **p value** | **Lower 95% Confidence interval** | **Upper 95% Confidence interval** | **Estimate** | **p value** | **Lower 95% Confidence interval** | **Upper 95% Confidence interval** |
| Sex female | -0.61 | 0.12 | -1.37 | 0.16 | 0.31 | 0.82 | -2.36 | 2.98 |
| Age >40 years | **-2.20** | **<0.001** | **-3.05** | **-1.35** | **-5.25** | **0.001** | **-8.29** | **-2.20** |
| >= 12 years of school education | **1.92** | **<0.001** | **1.13** | **2.70** | 1.60 | 0.30 | -1.37 | 4.57 |
| Smoking | **-1.89** | **0.007** | **-3.27** | **-0.51** | -0.17 | 0.94 | -4.36 | 4.01 |
| Obesity | **-1.56** | **0.002** | **-2.57** | **-0.55** | -1.95 | 0.24 | -5.20 | 1.29 |
| Medical treatment during index infection | **-1.97** | **<0.001** | **-2.88** | **-1.05** | -0.60 | 0.72 | -3.86 | 2.65 |
| Further SARS-CoV2-infection | 0.20 | 0.59 | -0.54 | 0.95 | **-3.45** | **0.010** | **-6.15** | **-0.74** |
| *Symptom clusters 24 months* |  |  |  |  |  |  |  |  |
| Fatigue | **-8.59** | **<0.001** | **-9.72** | **-7.46** | **-8.75** | **<0.001** | **-12.63** | **-4.87** |
| Neurocognitive impairment | **-9.50** | **<0.001** | **-10.66** | **-8.34** | **-10.51** | **<0.001** | **-14.50** | **-6.53** |
| Chest symptoms | **-3.29** | **<0.001** | **-4.56** | **-2.03** | **-6.96** | **0.001** | **-10.99** | **-2.94** |
| Musculoskeletal pain | **-3.36** | **<0.001** | **-4.58** | **-2.15** | -2.46 | 0.21 | -6.26 | 1.35 |
| Anxiety/depression/sleep disorders | **-6.11** | **<0.001** | **-7.22** | **-5.00** | **-6.07** | **0.001** | **-9.77** | **-2.38** |
| Smell/taste | **-2.36** | **0.002** | **-3.81** | **-0.90** | -1.82 | 0.47 | -6.74 | 3.10 |
| *Pre-existing comorbidities* |  |  |  |  |  |  |  |  |
| Musculoskeletal disorders | **-1.95** | **<0.001** | **-2.77** | **-1.13** | **-3.63** | **0.016** | **-6.57** | **-0.69** |
| Cardiovascular diseases | **-2.07** | **<0.001** | **-3.03** | **-1.10** | -3.02 | 0.073 | -6.33 | 0.29 |
| Respiratory diseases | **-1.47** | **0.006** | **-2.52** | **-0.42** | -0.17 | 0.92 | -3.56 | 3.23 |
| Mental disorders | **-7.20** | **<0.001** | **-8.26** | **-6.14** | **-9.13** | **<0.001** | **-12.61** | **-5.66** |

mWAI2: modified work ability index, task related work ability, Symptom clusters were not present before the SARS-CoV-2 infection, bold letters indicate statistically significant at p<0.05

**Supplementary Table 3E:** **Association between potential risk factors including symptom clusters measured 24 months after initial infection and task related work ability (mWAI2). Results of a mutually adjusted linear regression analysis stratified by working task among female participants.**

|  | **Female participants with mental working task (n=2959)** | | | | **Female participants with physical working task (n=261)** | | | |
| --- | --- | --- | --- | --- | --- | --- | --- | --- |
| **Predictor** | **Estimate** | **p value** | **Lower 95% Confidence interval** | **Upper 95% Confidence interval** | **Estimate** | **p value** | **Lower 95% Confidence interval** | **Upper 95% Confidence interval** |
| Age >40 years | **-2.25** | **<0.001** | **-3.33** | **-1.17** | -3.81 | 0.094 | -8.26 | 0.65 |
| >= 12 years of school education | **1.66** | **0.001** | **0.66** | **2.65** | 2.90 | 0.16 | -1.15 | 6.94 |
| Smoking | **-2.56** | **0.004** | **-4.30** | **-0.83** | 1.05 | 0.71 | -4.55 | 6.64 |
| Obesity | -0.73 | 0.28 | -2.03 | 0.58 | -2.15 | 0.38 | -6.89 | 2.59 |
| Medical treatment during index infection | **-1.67** | **0.005** | **-2.83** | **-0.50** | -0.10 | 0.97 | -4.55 | 4.35 |
| Further SARS-CoV2-infection | 0.42 | 0.39 | -0.53 | 1.37 | -3.56 | 0.065 | -7.35 | 0.22 |
| *Symptom clusters 24 months* |  |  |  |  |  |  |  |  |
| Fatigue | **-8.09** | **<0.001** | **-9.45** | **-6.74** | **-7.89** | **0.002** | **-12.75** | **-3.03** |
| Neurocognitive impairment | **-8.93** | **<0.001** | **-10.32** | **-7.54** | **-10.55** | **<0.001** | **-16.05** | **-5.06** |
| Chest symptoms | **-2.92** | **<0.001** | **-4.44** | **-1.40** | -4.19 | 0.15 | -9.43 | 1.04 |
| Musculoskeletal pain | **-4.20** | **<0.001** | **-5.67** | **-2.74** | -1.84 | 0.49 | -7.05 | 3.36 |
| Anxiety/depression/sleep disorders | **-5.02** | **<0.001** | **-6.37** | **-3.68** | -3.65 | 0.16 | -8.77 | 1.47 |
| Smell/taste | **-2.42** | **0.006** | **-4.15** | **-0.69** | -2.46 | 0.45 | -8.80 | 3.88 |
| *Pre-existing comorbidities* |  |  |  |  |  |  |  |  |
| Musculoskeletal disorders | -0.86 | 0.11 | -1.90 | 0.18 | -3.01 | 0.15 | -7.13 | 1.11 |
| Cardiovascular diseases | **-2.21** | **0.001** | **-3.50** | **-0.93** | -1.94 | 0.43 | -6.78 | 2.91 |
| Respiratory diseases | **-1.91** | **0.004** | **-3.22** | **-0.61** | -1.80 | 0.47 | -6.71 | 3.10 |
| Mental disorders | **-6.23** | **<0.001** | **-7.50** | **-4.96** | **-9.21** | **<0.001** | **-13.82** | **-4.61** |

mWAI2: modified work ability index, task related work ability, Symptom clusters were not present before the SARS-CoV-2 infection, bold letters indicate statistically significant at p<0.05

**Supplementary Table 3F:** **Association between potential risk factors including symptom clusters measured 24 months after initial infection and task related work ability (mWAI2). Results of a mutually adjusted linear regression analysis stratified by working task among male participants.**

|  | **Male participants with mental working task (n=1939)** | | | | **Male participants with physical working task (n=188)** | | | |
| --- | --- | --- | --- | --- | --- | --- | --- | --- |
| **Predictor** | **Estimate** | **p value** | **Lower 95% Confidence interval** | **Upper 95% Confidence interval** | **Estimate** | **p value** | **Lower 95% Confidence interval** | **Upper 95% Confidence interval** |
| Age >40 years | **-2.13** | **0.002** | **-3.50** | **-0.76** | **-6.05** | **0.005** | **-10.28** | **-1.83** |
| >= 12 years of school education | **2.17** | **<0.001** | **0.92** | **3.43** | 0.22 | 0.93 | -4.46 | 4.90 |
| Smoking | -0.96 | 0.40 | -3.21 | 1.29 | -2.52 | 0.45 | -9.17 | 4.12 |
| Obesity | **-2.83** | **<0.001** | **-4.41** | **-1.26** | -0.44 | 0.85 | -4.96 | 4.07 |
| Medical treatment during index infection | **-2.47** | **0.001** | **-3.95** | **-1.00** | -1.09 | 0.67 | -6.17 | 3.99 |
| Further SARS-CoV2-infection | -0.26 | 0.67 | -1.44 | 0.93 | -2.55 | 0.21 | -6.53 | 1.44 |
| *Symptom clusters 24 months* |  |  |  |  |  |  |  |  |
| Fatigue | **-10.09** | **<0.001** | **-12.12** | **-8.06** | **-8.16** | **0.029** | **-15.35** | **-0.98** |
| Neurocognitive impairment | **-10.27** | **<0.001** | **-12.33** | **-8.21** | **-9.97** | **0.002** | **-16.24** | **-3.70** |
| Chest symptoms | **-3.70** | **0.001** | **-5.94** | **-1.45** | **-11.47** | **0.001** | **-18.38** | **-4.55** |
| Musculoskeletal pain | -1.80 | 0.101 | -3.95 | 0.35 | -2.80 | 0.33 | -8.50 | 2.91 |
| Anxiety/depression/sleep disorders | **-8.19** | **<0.001** | **-10.10** | **-6.29** | **-11.54** | **<0.001** | **-17.24** | **-5.84** |
| Smell/taste | -2.35 | 0.082 | -5.00 | 0.30 | 0.18 | 0.97 | -8.55 | 8.91 |
| *Pre-existing comorbidities* |  |  |  |  |  |  |  |  |
| Musculoskeletal disorders | **-3.59** | **<0.001** | **-4.91** | **-2.26** | **-6.18** | **0.007** | **-10.60** | **-1.76** |
| Cardiovascular diseases | **-1.76** | **0.017** | **-3.20** | **-0.32** | -3.80 | 0.10 | -8.36 | 0.77 |
| Respiratory diseases | -0.78 | 0.38 | -2.52 | 0.96 | 1.30 | 0.61 | -3.74 | 6.34 |
| Mental disorders | **-9.66** | **<0.001** | **-11.56** | **-7.77** | **-8.78** | **0.003** | **-14.43** | **-3.14** |

mWAI2: modified work ability index, task related work ability, Symptom clusters were not present before the SARS-CoV-2 infection, bold letters indicate statistically significant at p<0.05

**Supplementary Table 3G:** **Association between potential risk factors including symptom clusters reported 24 months after initial infection and task related work ability (mWAI2). Results of a mutually adjusted linear regression analysis stratified by comorbid mental disorders.**

|  | **No comorbid mental disorders (n=4317)** | | | | **Comorbid mental disorders (n=922)** | | | |
| --- | --- | --- | --- | --- | --- | --- | --- | --- |
| **Predictor** | **Estimate** | **p value** | **Lower 95% Confidence interval** | **Upper 95% Confidence interval** | **Estimate** | **p value** | **Lower 95% Confidence interval** | **Upper 95% Confidence interval** |
| Sex female | **-1.18** | **0.003** | **-1.94** | **-0.41** | **3.37** | **0.004** | **1.10** | **5.64** |
| Age >40 years | **-2.13** | **<0.001** | **-2.98** | **-1.29** | **-3.87** | **0.004** | **-6.46** | **-1.28** |
| >= 12 years of school education | **2.27** | **<0.001** | **1.48** | **3.06** | -0.40 | 0.72 | -2.62 | 1.82 |
| Smoking | -0.81 | 0.26 | -2.23 | 0.61 | **-4.58** | **0.007** | **-7.88** | **-1.27** |
| Obesity | **-1.76** | **<0.001** | **-2.80** | **-0.73** | -1.08 | 0.39 | -3.56 | 1.40 |
| Physical working task | **-1.57** | **0.029** | **-2.98** | **-0.16** | **-4.70** | **0.009** | **-8.21** | **-1.20** |
| Medical treatment during index infection | **-2.26** | **<0.001** | **-3.21** | **-1.32** | -0.19 | 0.87 | -2.50 | 2.12 |
| Further SARS-CoV2-infection | -0.24 | 0.53 | -0.99 | 0.51 | 0.36 | 0.73 | -1.73 | 2.46 |
| *Symptom clusters 24 months* |  |  |  |  |  |  |  |  |
| Fatigue | **-8.78** | **<0.001** | **-10.00** | **-7.57** | **-8.91** | **<0.001** | **-11.40** | **-6.42** |
| Neurocognitive impairment | **-9.54** | **<0.001** | **-10.80** | **-8.29** | **-9.75** | **<0.001** | **-12.26** | **-7.24** |
| Chest symptoms | **-3.08** | **<0.001** | **-4.45** | **-1.72** | **-5.37** | **<0.001** | **-8.08** | **-2.66** |
| Musculoskeletal pain | **-3.74** | **<0.001** | **-5.03** | **-2.45** | -2.09 | 0.13 | -4.75 | 0.58 |
| Anxiety/depression/sleep disorders | **-7.11** | **<0.001** | **-8.27** | **-5.94** | **-3.08** | **0.019** | **-5.64** | **-0.52** |
| Smell/taste | **-2.86** | **<0.001** | **-4.40** | **-1.32** | -0.93 | 0.58 | -4.22 | 2.36 |
| *Pre-existing comorbidities* |  |  |  |  |  |  |  |  |
| Musculoskeletal disorders | **-1.92** | **<0.001** | **-2.76** | **-1.08** | **-2.45** | **0.031** | **-4.67** | **-0.22** |
| Cardiovascular diseases | **-2.30** | **<0.001** | **-3.29** | **-1.31** | -1.92 | 0.12 | -4.36 | 0.51 |
| Respiratory diseases | **-1.13** | **0.048** | **-2.26** | **-0.01** | -2.12 | 0.068 | -4.39 | 0.16 |

mWAI2: modified work ability index, task related work ability, Symptom clusters were not present before the SARS-CoV-2 infection, bold letters indicate statistically significant at p<0.05

**Supplementary Table 3H:** **Association between potential risk factors including symptom clusters reported 24 months after initial infection and task related work ability (mWAI2). Results of a mutually adjusted linear regression analysis stratified by comorbid mental disorders among female participants.**

|  | **Female participants, no comorbid mental disorders**  **(n=2505)** | | | | **Female participants, comorbid mental disorders (n=641)** | | | |
| --- | --- | --- | --- | --- | --- | --- | --- | --- |
| **Predictor** | **Estimate** | **p value** | **Lower 95% Confidence interval** | **Upper 95% Confidence interval** | **Estimate** | **p value** | **Lower 95% Confidence interval** | **Upper 95% Confidence interval** |
| Age >40 years | **-1.66** | **0.003** | **-2.77** | **-0.55** | **-5.27** | **<0.001** | **-8.14** | **-2.40** |
| >= 12 years of school education | **2.50** | **<0.001** | **1.46** | **3.54** | -1.36 | 0.29 | -3.87 | 1.16 |
| Smoking | -1.20 | 0.20 | -3.05 | 0.65 | **-4.21** | **0.025** | **-7.90** | **-0.53** |
| Obesity | **-1.41** | **0.049** | **-2.81** | **-0.01** | 0.64 | 0.65 | -2.17 | 3.45 |
| Physical working task | -1.26 | 0.18 | -3.11 | 0.58 | **-4.92** | **0.019** | **-9.03** | **-0.81** |
| Medical treatment during index infection | **-1.73** | **0.006** | **-2.96** | **-0.50** | -0.68 | 0.62 | -3.37 | 2.00 |
| Further SARS-CoV2-infection | -0.03 | 0.95 | -1.02 | 0.96 | 0.51 | 0.67 | -1.89 | 2.92 |
| *Symptom clusters 24 months* |  |  |  |  |  |  |  |  |
| Fatigue | **-8.22** | **<0.001** | **-9.71** | **-6.73** | **-7.90** | **<0.001** | **-10.66** | **-5.13** |
| Neurocognitive impairment | **-8.90** | **<0.001** | **-10.45** | **-7.35** | **-9.43** | **<0.001** | **-12.26** | **-6.61** |
| Chest symptoms | **-2.60** | **0.003** | **-4.28** | **-0.91** | **-4.78** | **0.002** | **-7.79** | **-1.77** |
| Musculoskeletal pain | **-4.43** | **<0.001** | **-6.03** | **-2.83** | **-3.20** | **0.035** | **-6.18** | **-0.22** |
| Anxiety/depression/sleep disorders | **-6.39** | **<0.001** | **-7.86** | **-4.93** | -0.69 | 0.64 | -3.57 | 2.18 |
| Smell/taste | **-3.06** | **0.002** | **-4.94** | **-1.17** | -0.72 | 0.69 | -4.28 | 2.83 |
| *Pre-existing comorbidities* |  |  |  |  |  |  |  |  |
| Musculoskeletal disorders | **-1.30** | **0.020** | **-2.39** | **-0.21** | 0.93 | 0.47 | -1.59 | 3.44 |
| Cardiovascular diseases | **-2.34** | **0.001** | **-3.72** | **-0.95** | -2.16 | 0.13 | -4.92 | 0.61 |
| Respiratory diseases | **-1.51** | **0.041** | **-2.95** | **-0.06** | **-3.25** | **0.016** | **-5.89** | **-0.61** |

mWAI2: modified work ability index, task related work ability, Symptom clusters were not present before the SARS-CoV-2 infection, bold letters indicate statistically significant at p<0.05

**Supplementary Table 3I:** **Association between potential risk factors including symptom clusters reported 24 months after initial infection and task related work ability (mWAI2). Results of a mutually adjusted linear regression analysis stratified by comorbid mental disorders among male participants.**

|  | **Male participants, no comorbid mental disorders (n=1812)** | | | | **Male participants, comorbid mental disorders (n=281)** | | | |
| --- | --- | --- | --- | --- | --- | --- | --- | --- |
| **Predictor** | **Estimate** | **p value** | **Lower 95% Confidence interval** | **Upper 95% Confidence interval** | **Estimate** | **p value** | **Lower 95% Confidence interval** | **Upper 95% Confidence interval** |
| Age >40 years | **-2.77** | **<0.001** | **-4.08** | **-1.45** | 1.15 | 0.68 | -4.34 | 6.63 |
| >= 12 years of school education | **1.97** | **0.002** | **0.74** | **3.20** | 1.36 | 0.54 | -3.02 | 5.75 |
| Smoking | -0.29 | 0.80 | -2.51 | 1.93 | -5.31 | 0.12 | -12.10 | 1.48 |
| Obesity | **-2.20** | **0.005** | **-3.74** | **-0.65** | **-5.33** | **0.036** | **-10.32** | **-0.35** |
| Physical working task | -2.12 | 0.058 | -4.30 | 0.07 | -4.06 | 0.21 | -10.47 | 2.36 |
| Medical treatment during index infection | **-2.86** | **<0.001** | **-4.34** | **-1.37** | -0.25 | 0.91 | -4.62 | 4.11 |
| Further SARS-CoV2-infection | -0.57 | 0.34 | -1.74 | 0.60 | -0.47 | 0.82 | -4.58 | 3.64 |
| *Symptom clusters 24 months* |  |  |  |  |  |  |  |  |
| Fatigue | **-9.85** | **<0.001** | **-11.95** | **-7.75** | **-12.33** | **<0.001** | **-17.68** | **-6.98** |
| Neurocognitive impairment | **-10.63** | **<0.001** | **-12.76** | **-8.49** | **-8.53** | **0.001** | **-13.71** | **-3.34** |
| Chest symptoms | **-3.81** | **0.001** | **-6.15** | **-1.47** | **-6.40** | **0.025** | **-12.00** | **-0.80** |
| Musculoskeletal pain | **-2.84** | **0.011** | **-5.03** | **-0.64** | 1.41 | 0.62 | -4.19 | 7.01 |
| Anxiety/depression/sleep disorders | **-8.39** | **<0.001** | **-10.32** | **-6.46** | **-9.58** | **<0.001** | **-14.71** | **-4.44** |
| Smell/taste | -2.52 | 0.064 | -5.18 | 0.15 | -0.07 | 0.97 | -7.60 | 7.46 |
| *Pre-existing comorbidities* |  |  |  |  |  |  |  |  |
| Musculoskeletal disorders | **-2.81** | **<0.001** | **-4.12** | **-1.51** | **-10.28** | **<0.001** | **-14.72** | **-5.84** |
| Cardiovascular diseases | **-2.11** | **0.004** | **-3.52** | **-0.69** | -0.01 | 0.98 | -4.96 | 4.94 |
| Respiratory diseases | -0.67 | 0.47 | -2.46 | 1.13 | -0.84 | 0.80 | -5.07 | 3.40 |

mWAI2: modified work ability index, task related work ability, Symptom clusters were not present before the SARS-CoV-2 infection, bold letters indicate statistically significant at p<0.05

**Supplementary Table 4:** **Association between potential risk factors including symptom clusters reported 24 months after initial infection and weeks of sick leave. Results of a mutually adjusted linear regression analysis (n=5422).**

| **Predictor** | **Estimate** | **p value** | **Lower 95% Confidence interval** | **Upper 95% Confidence interval** |
| --- | --- | --- | --- | --- |
| Sex female | **-0.78** | **0.008** | **-1.36** | **-0.20** |
| Age >40 years | -0.09 | 0.79 | -0.73 | 0.56 |
| >= 12 years of school education | 0.18 | 0.56 | -0.42 | 0.77 |
| Smoking | -0.49 | 0.35 | -1.53 | 0.54 |
| Obesity | -0.02 | 0.97 | -0.77 | 0.74 |
| Physical working task | 0.06 | 0.91 | -0.98 | 1.10 |
| Medical treatment during index infection | 0.51 | 0.15 | -0.18 | 1.21 |
| Further SARS-CoV2-infection | **-0.61** | **0.036** | **-1.17** | **-0.04** |
| *Symptom clusters 24 months* |  |  |  |  |
| Fatigue | **2.74** | **<0.001** | **1.89** | **3.60** |
| Neurocognitive impairment | **1.45** | **0.001** | **0.57** | **2.33** |
| Chest symptoms | **1.23** | **0.012** | **0.27** | **2.19** |
| Musculoskeletal pain | **2.43** | **<0.001** | **1.52** | **3.35** |
| Anxiety/depression/sleep disorders | 0.55 | 0.20 | -0.29 | 1.38 |
| Smell/taste | -0.11 | 0.87 | -1.21 | 1.00 |
| *Pre-existing comorbidities* |  |  |  |  |
| Musculoskeletal disorders | -0.37 | 0.25 | -0.99 | 0.25 |
| Cardiovascular diseases | **0.98** | **0.008** | **0.25** | **1.71** |
| Respiratory diseases | 0.45 | 0.26 | -0.34 | 1.24 |
| Mental disorders | **2.77** | **<0.001** | **1.97** | **3.57** |

Symptom clusters were not present before the SARS-CoV-2 infection, bold letters indicate statistically significant at p<0.05

**Supplementary Table 5:** **Association between potential risk factors including post-exertional malaise or ME/CFS and task related work ability (mWAI2). Results of a mutually adjusted linear regression analysis (n=5422).**

|  | **Model including post-exertional malaise** | | | | **Model including ME/CFS** | | | |
| --- | --- | --- | --- | --- | --- | --- | --- | --- |
| **Predictor** | **Estimate** | **p value** | **Lower 95% Confidence interval** | **Upper 95% Confidence interval** | **Estimate** | **p value** | **Lower 95% Confidence interval** | **Upper 95% Confidence interval** |
| Sex female | **-1.93** | **<0.001** | **-2.69** | **-1.16** | **-2.58** | **<0.001** | **-3.57** | **-1.59** |
| Age >40 years | **-4.74** | **<0.001** | **-5.59** | **-3.90** | **-4.61** | **<0.001** | **-5.71** | **-3.51** |
| >= 12 years of school education | **2.53** | **<0.001** | **-3.32** | **-1.73** | **3.54** | **<0.001** | **-4.55** | **-2.52** |
| Smoking | **-2.43** | **0.001** | **-3.80** | **-1.06** | **-3.85** | **<0.001** | **-5.54** | **-2.15** |
| Obesity | **-1.92** | **<0.001** | **-2.91** | **-0.94** | **-4.51** | **<0.001** | **-5.71** | **-3.30** |
| Physical working task | **-3.34** | **<0.001** | **-4.70** | **-1.97** | **-2.64** | **0.003** | **-4.35** | **-0.93** |
| Medical treatment during index infection | **-3.41** | **<0.001** | **-4.09** | **-2.73** | **-5.07** | **<0.001** | **-5.90** | **-4.24** |
| Further SARS-CoV2-infection | -0.33 | 0.40 | -1.09 | 0.43 | -0.68 | 0.17 | -1.66 | 0.29 |
| Post-exertional malaise# | **-20.69** | **<0.001** | **-21.66** | **-19.72** | - | - | - | - |
| ME/CFS# | - | - | - | - | **-21.14** | **<0.001** | **-23.19** | **-19.08** |

mWAI2: modified work ability index, task related work ability, bold letters indicate statistically significant at p<0.05, ME/CFS: Myalgic Encephalomyelitis/Chronic Fatigue Syndrome, #in the model with post-exertional malaise we did not adjust for ME/CFS and vice versa
